# Experimental data of CaTiO_3_ photocatalyst for degradation of organic pollutants (Brilliant green dye) – Green synthesis, characterization and kinetic study

**DOI:** 10.1016/j.dib.2020.106099

**Published:** 2020-07-31

**Authors:** Lusi Ernawati, Ruri Agung Wahyuono, Hendri Widiyandari, Doty Dewi Risanti, Ade Wahyu Yusariarta, Rebeka, Virginia Sitompul

**Affiliations:** a Department of Chemical Engineering, Institut Teknologi Kalimantan, Balikpapan 76127, Indonesia; b Department of Engineering Physics, Institut Teknologi Sepuluh Nopember, Surabaya 60111, Indonesia; c Department of Physics, Universitas Sebelas Maret, Surakarta 57126, Indonesia; d Department of Materials and Metallurgical Engineering, Institut Teknologi Kalimantan, Balikpapan 76127, Indonesia

**Keywords:** Perovskite, Calcium titanate, Chicken eggshells, Anatase TiO_2_, UV photoreactor, Kinetics

## Abstract

The data presented here focuses on the physicochemical characterization of perovskite CaTiO_3_ nanoparticles (orthorhombic) as photocatalyts and the kinetic study of their photodegradation performance toward organic pollutant, i.e. brilliant green (BG) which is azo derivatives dye. The CaTiO_3_ nanoparticles was synthesized using chicken eggshell-derived CaCO_3_ and anatase TiO_2_ with molar ratio of (1:1), (1:3), (2:5), and (2:7). The physical and microstructural properties of CaTiO_3_ were characterized by X-ray diffractometer (XRD), scanning electron microscope (SEM), Fourier Transform Infrared (FTIR) and UV/vis spectrometer. The effect of initial dye concentration, catalyst composition, and catalyst dosage on the adsorption mechanism of dye on CaTiO_3_ was investigated in jacketed photoreactor under UV irradiation. The analysis reveals that BG molecules are efficiently chemisorbed, as indicated by *pseudo* first order kinetic, and degraded within 120 min. Considering the low-cost preparation process and high photocatalytic performance, the resultant CaTiO_3_ can further be used as an efficient photocatalyst for organic pollutant removal from aqueous and industrial wastewater.

**Specifications Table**

**Table:** 

**Subject**	Materials Chemistry
**Specific subject area**	Photocatalysis
**Type of data**	Table, Image, Graph
**How data were acquired**	CaTiO_3_ powder was prepared using wet chemical synthesis, in which chicken eggshells as precursor source were collected from the farm field in Samboja, Balikpapan, Indonesia. Physicochemical characterizations were carried out by scanning electron microscope (SEM), X-ray diffractometer (XRD), Fourier Transform Infrared (FTIR) spectrometer. The kinetic data was fitted using both *pseudo* first order and *pseudo* second order adsorption model.
**Data format**	Raw and Analyzed
**Parameters for data collection**	X-ray diffractometer was operated at 40 kV, and 40 mA with Cu-Kα as a radiation source. Diffraction patterns were scanned between 10 and 100° (2θ) with resolutions of 0.05° FTIR spectra were collected in wavenumber range between 400 and 4000 cm^−1^. SEM images were collected at 100 kV accelerating voltage with 500 × magnification. UV photoreactor was filled with 10 ppm of brilliant green solution and run under continuous stirring (500 rpm) at 28 °C.
**Description of data collection**	Morphology of CaTiO_3_ was assessed using SEM (FEI Inspect 21). XRD patterns were collected using a diffractometer (PAN analytical type X'Pert Pro). FTIR spectra were recorded using Thermo Nicole is50 spectrometer at room temperature. Degradation of aqueous brilliant green (BG) dyes was probed under UV photoreactor using simulated UV irradiation (T5-UV7W, 254 nm). UV/vis absorption spectra to probe degradation of brilliant green dye were measured using UV/vis spectrometer (Rayleigh UV-9200).
**Data source location**	Department of Chemical Engineering, Institut Teknologi Kalimantan, Balikpapan, East Kalimantan, Indonesia (−1.135330, 116.858093) Department of Engineering Physics, Institut Teknologi Sepuluh Nopember, Surabaya, East Java, Indonesia (−7.283395, 112.795727) Central Laboratory, State University Malang, East Java, Indonesia (−7.961229, 112.618759)
**Data accessibility**	Data are available within the article

**Value of the Data**

The CaTiO_3_ nanomaterial investigated here renders perovskite based photocatalyst which has been proven its functionality for photocatalytic degradation of azo dyes derivative, i.e. brilliant green (BG).
The current data, particularly the kinetic of degradation of aqueous BG solution, is useful for relevant studies of photocatalytic azo dye degradation using other catalysts, which is not limited to pristine CaTiO_3_, CaCO_3_, TiO_2_ or composite materials. The photocatalytic degradation data suggest that the current prepared CaTiO_3_ materials can be readily utilized as photocatalyst for wastewater treatment in textile industry, food processing industry, and for water treatment in water utility company.
The physicochemical data highlights the current synthesis route could not yield 100% CaTiO_3_ and hence, optimization of precursor composition and mechanochemical as well as post heat treatment will be the focus of further research.
The preparation of CaTiO_3_ nanomaterial investigated here is considered low cost and green since the wet chemical synthetic route didn't require sophisticated apparatus while the precursor employed chicken eggshells (waste or by-product of farming activities).

## 1 Data description

**Fig. 1:**
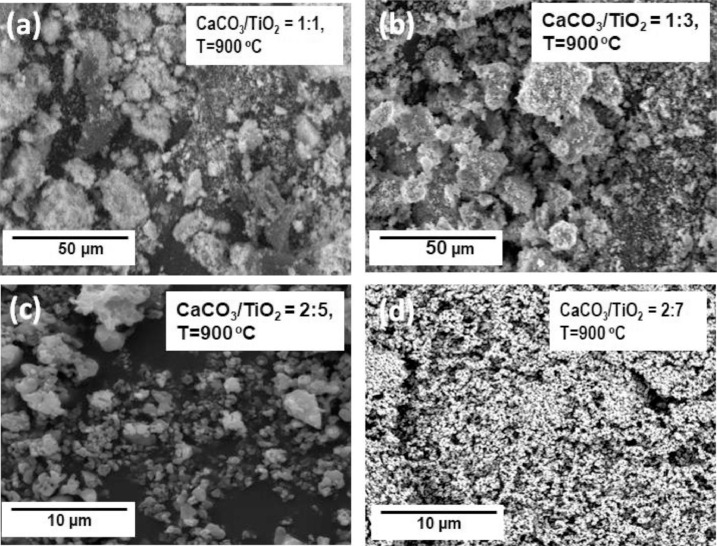
Scanning electron micrographs of CaTiO_3_ prepared using different CaCO_3_/TiO_2_ molar ratio, i.e. (a) (1:1), (b) (1:3), and (c) (2:5). All samples were annealed at 900 °C for 4 h.

**Fig. 2:**
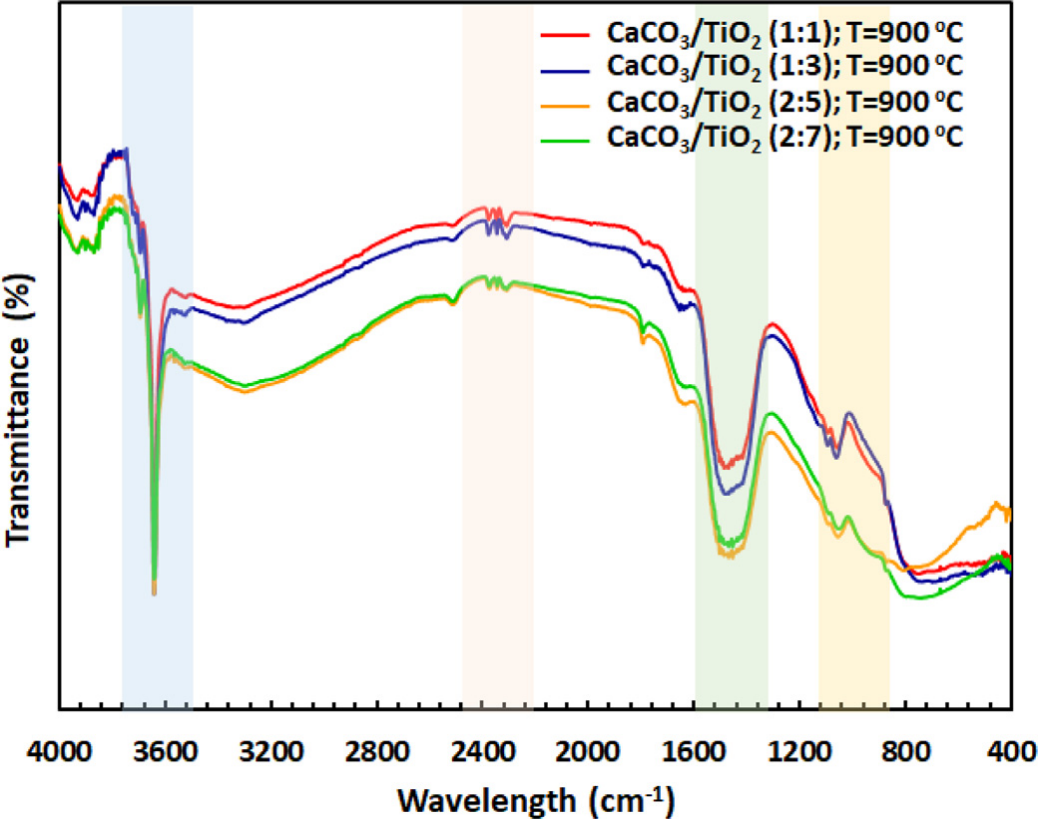
FTIR spectra of CaTiO_3_ prepared with different CaCO_3_/TiO_2_ molar ratio.

**Fig. 3:**
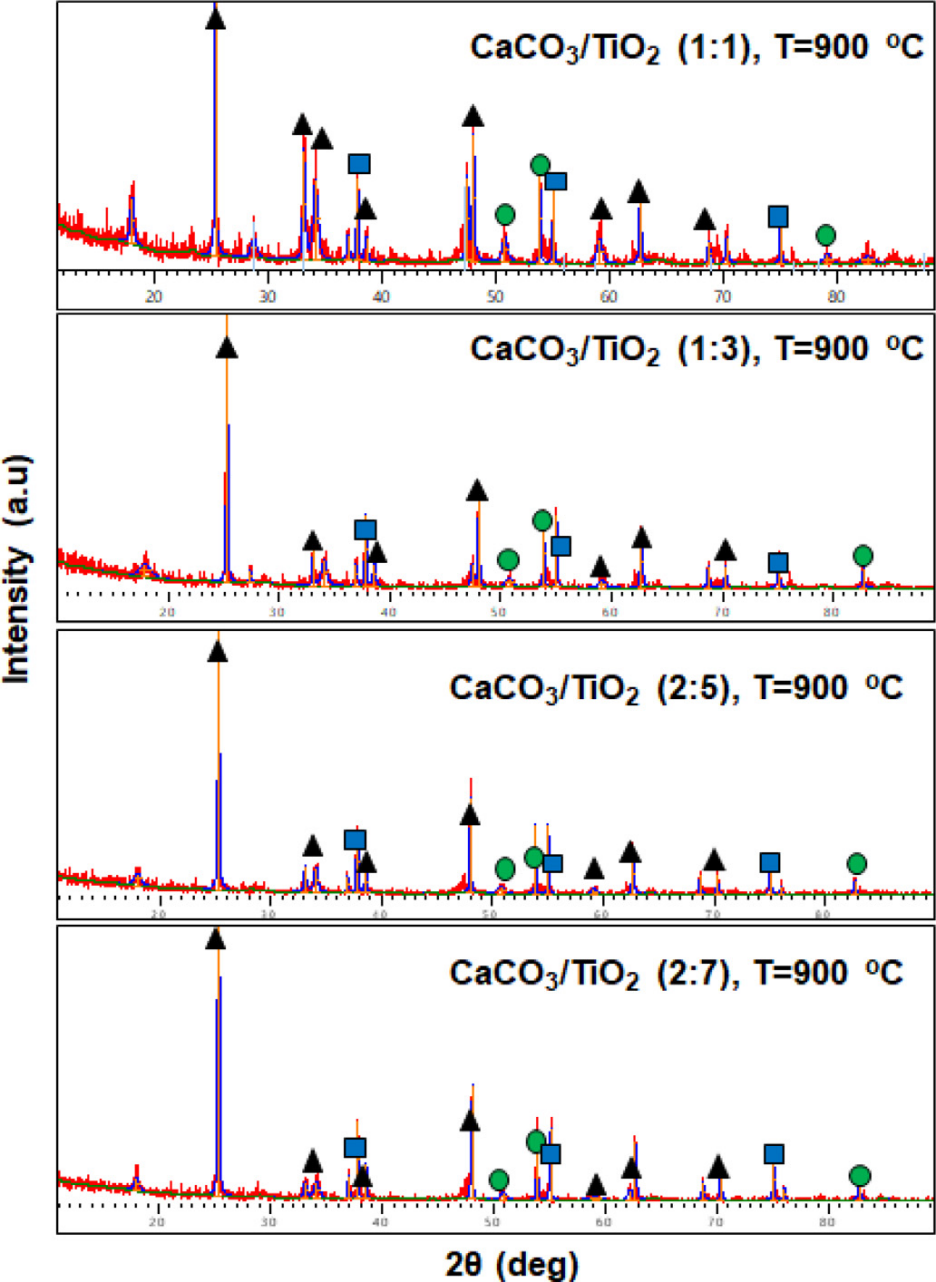
X-ray diffraction pattern of CaTiO_3_ prepared using different CaCO_3_/TiO_2_ molar ratio. Triangle (▲), square ( ▀) and circle (☻) denote CaTiO_3_, CaCO_3_, and TiO_2_, respectively.

Physicochemical characteristics of various CaTiO_3_ are evaluated from the raw data, including scanning electron micrograph, X-ray diffraction pattern and FTIR spectra (available in the Supplementary Material). The surface morphology of different nanostructured CaTiO_3_ prepared using different CaCO_3_/TiO_2_ molar ratio are depicted in Fig. 1. Higher TiO_2_ fraction breaks the aggregation formed in CaTiO_3_ prepared using large fraction of CaCO_3_ due to higher surface energy of TiO_2_ (1.4 × 10^7^ erg/cm^2^) than that of CaCO_3_ (1.7 × 10^4^ erg/cm^2^) [1,2]. Electronic vibrational characteristics and microstructural properties are indicated by FTIR spectra (Fig. 2) and X-ray diffraction pattern (Fig. 3), respectively. The decreasing IR bands at ∼3630 cm^−1^ and ∼1440 cm^−1^ are associated with the vibration characteristics of the hydroxy (OH) group and symmetric as well as asymmetric vibration between metal oxides, respectively [3]. In addition, the decreasing signal amplitude at ∼1150 cm^−1^ associated with C—O-Ti group vibration upon increasing TiO_2_ mol fraction might indicate the more efficient interconversion into Ca-O-Ti reflected by higher absorption at ∼660 cm^−1^ [4]. XRD patterns indicate the formation orthorhombic CaTiO_3_ with the presence of excess precursors, i.e. CaCO_3_ and TiO_2_. The diffraction peaks at 2θ of 23.2°, 33.1°, 47.5°, 58.8°, and 59.3° are assigned to the crystal planes of (101), (121), (202), (321), and (123), respectively [5]. Increasing the TiO_2_ fraction from CaCO_3_/TiO_2_ molar ratio in the preparation of CaTiO_3_ nanoparticles inreases the crystallite size, i.e. 17.7, 22.9, 34,6, and 37.2 nm for (1:1), (1:3), (2:5), and (2:7), respectively. This implies that the specific surface area decreases upon increasing TiO_2_ molar fraction.

**Fig. 4:**
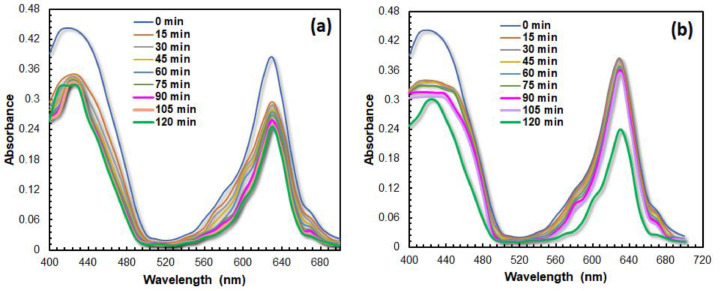
The time dependent absorption spectra of BG (10 ppm) upon photodegradation using CaTiO_3_ with CaCO_3_/TiO_2_ molar ratio of (a) (1:3) and (b) (2:7). The amount of catalyst was fixed at 50 mg.

**Fig. 5:**
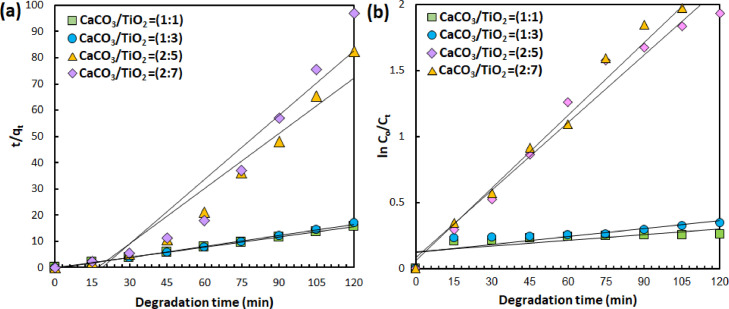
(a) *Pseudo* second order and (b) *pseudo* first order kinetic fit from photodegradation data of 10 ppm BG employing CaTiO_3_ prepared from different CaCO_3_/TiO_2_ composition.

**Fig. 6:**
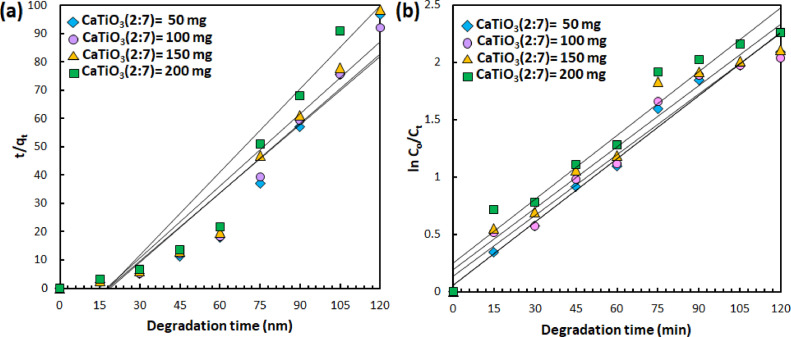
(a) *Pseudo* second order and (b) *pseudo* first order kinetic fit from photodegradation data of 10 ppm BG employing CaTiO_3_ (2:7) with different catalyst dosages.

**Fig. 7:**
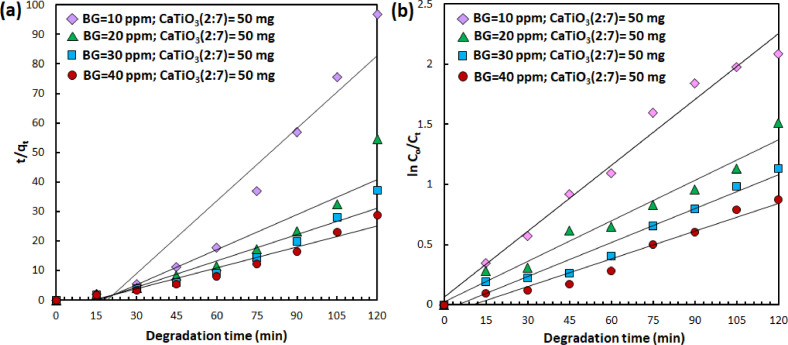
(a) *Pseudo* second order and (b) *pseudo* first order kinetic fit from photodegradation data of different BG concentration employing 50 mg CaTiO_3_ (2:7).

Having characterized the physicochemical properties, the photocatalytic degradation of aqueous BG dyes using the resulting CaTiO_3_ catalyst were investigated by probing the temporal change of UV/vis absorption spectra (Fig. 4, representative/selected raw data is available in the Supplementary Material). Kinetic of degradation mechanism to understand the adsorption process of dye molecules toward catalyst surface is evaluated using both *pseudo* first order and *pseudo* second order kinetic fit (Fig. 5, Fig. 6, Fig. 7). For *pseudo* first order fit, a plot of ln(C_o_/C_t_) vs *t* (C_0_ and C_t_ denote concentration at initial condition and time t, respectively) results in a linear curve, in which the slope equals to the observed rate constant (K_1_) [6]. Meanwhile, *pseudo* second order fit, the off-set of the linear plot of t/q_e_ vs t, where q_e_ is the concentration at equilibrium condition, yield the rate constant (K_2_) [6]. The rate constant of photocatalytic degradation upon varying the catalyst composition, catalyts dosage and pollutant concentration is summarized in Table 1, Table 2, Table 3. The analysis indicates that increasing the fraction of TiO_2_ in the precursor composition, i.e. CaCO_3_/TiO_2_ molar ratio, alters the adsorption behavior from physisorption (following second order reaction, R^2^ > 0.9) to chemisorption (following first order reaction, R^2^ > 0.9). In addition, increasing amount of CaTiO_3_ catalysts implies on the increasing chemisorbed BG molecules and faster catalytic reaction. Even though the degradation rate is slower than the highest reported in literature using CaTiO_3_ [7], [8], [9], the photodegradation rate of BG molecules using CaTiO_3_ in this work (0.0185 ppm•min^−1^) is found comparable to the photodegradation rate of other organic dye pollutants using solvothermal prepared CaTiO_3_ (0.162 ppm•min^−1^) [8] and hydrothermally prepared CaTiO_3_ (0.05•ppm⋅min^−1^) [9].

**Table 1:** Reaction rate constants (K) derived from both pseudo first order (K_1_) and pseudo second order (K_2_) as well as the corresponding coefficient of determination (R^2^) obtained for photodegradation of BG using different CaTiO_3_ composition.

CaCO_3_/TiO_2_ Molar Ratio	*K_1_* (min^−1^)	R^2^	*K_2_* (min^−1^)	R^2^
(1:1)	0.0014	0.5153	0.1301	0.9718
(1:3)	0.0023	0.6834	0.1403	0.9771
(2:5)	0.0176	0.9687	0.7022	0.9322
(2:7)	0.0183	0.9818	0.8185	0.9063

**Table 2:** Reaction rate constants (K) derived from both pseudo first order (K_1_) and pseudo second order (K_2_) as well as the corresponding coefficient of determination (R^2^) obtained for photodegradation of BG using different amount of CaTiO_3_ (2:7).

CaTiO_3_(2:7) Dosage (mg)	*K_1_* (min^−1^)	R^2^	*K_2_* (min^−1^)	R^2^
50	0.0185	0.9502	0.8185	0.9061
100	0.0183	0.9818	0.8032	0.9198
150	0.0178	0.9518	0.8491	0.9247
200	0.0176	0.9626	0.9802	0.95153

**Table 3:** Reaction rate constants (K) derived from both pseudo first order (K_1_) and pseudo second order (K_2_) as well as the corresponding coefficient of determination (R^2^) obtained for photodegradation of various BG concentration using 50 mg of CaTiO_3_ (2:7).

BG Concentration (ppm)	*K_1_* (min^−1^)	R^2^	*K_2_* (min^−1^)	R^2^
10	0.0183	0.9818	0.8185	0.9061
20	0.0113	0.9696	0.3971	0.8588
30	0.0094	0.9664	0.2984	0.9185
40	0.0076	0.9571	0.2363	0.9394

## 2 Experimental design, materials, and methods

Synthesis of CaTiO_3_ nanoparticles employed a precursor mixture of commercial anatase TiO_2_ (MTI, 99%) and CaCO_3_ extracted from eggshells [9]. The collected chicken eggshells were washed thoroughly with ethanol-water mixture and dried at ambient atmosphere for 48 h. Afterwards, the dried eggshells were grounded for 30 min into fine particles. The fine powder was treated with 0.1 M HCl for 1 h and then washed with distilled water prior heat treatment at 100 °C for 3 h. The heat-treated samples were then sieved (300 mesh) to yield fined CaCO_3_ nanoparticles (up to 86.6%). The initial step for CaTiO_3_ synthesis was to prepare a mixture of CaCO_3_ and TiO_2_ in different CaCO_3_/TiO_2_ molar ratio of (1:1), (1:3), (2:5), and (2:7), which was dissolved in 100 ml of ethanol and homogenized by continuous stirring at 300 rpm for 2 h at room temperature. The suspension was filtered and washed with distilled water several times and dried in an oven at 100 °C for 2 h. The dried white powder was subsequently grounded into fine and homogeneous granules, and eventually annealed at 900 °C for 4 h. Characterization of CaTiO_3_ nanoparticles follows the description in the specifications table (vide supra*)*.

Initial investigation of 10 ppm brilliant green (BG) photodegradation was carried out employing different CaTiO_3_ composition, i.e. different CaCO_3_/TiO_2_ molar ratio, in a custom-made photoreactor under UV irradiation [10], [11]. Additionally, the dosage of CaTiO_3_ and the initial concentration of BG solution were varied. It should be noted that the reactor was isolated from ambient light so that the photodegradation was driven only by UV irradiation. The solution in photoreactor was also continuously stirred to increase contact between CaTiO_3_ photocatalyst and BG molecules and hence, driving a rapid photodegradation. Photodegradation of BG was monitored through the absorption change (300 nm < λ < 800 nm) measured using UV/vis spectrometer. The decrease of BG optical density was used to determine the decreasing BG concentration due to the catalytic activity of CaTiO_3_, which was later used to evaluate the adsorption kinetics.

## Declaration of Competing Interest

The authors declare that they have no known competing financial interests or personal relationships that could have appeared to influence the work reported in this paper.

## Appendix Supplementary materials

Image, application 1

Image, application 2
