# Supplementary material for: Experimental data of CaTiO3 photocatalyst for degradation of organic pollutants (Brilliant green dye) – Green synthesis, characterization and kinetic study
Source: Data Brief. 2020 Jul 31;32:106099. doi: 10.1016/j.dib.2020.106099 (PMC7451799; doi:10.1016/j.dib.2020.106099)
Supplement: Supplementary file 1 [file mmc1.zip › All RAW Data for Data in Brief/XRD/XRD_CaTiO3 (1_1).pdf]

Anchor Scan Parameters

Dataset Name:	C211
File name:	E:\X'Pert Data\2020\Februari\20 Jan 2020\C211\C211.xrdml
Sample Identification:	C211
Comment:	Theta (10-90)<br>Configuration=Stage Flat Samples, Owner=User-1, Creation date=9/15/2009 2:20:30 PM<br>Goniometer=PW3050/60 (Theta/Theta); Minimum step size 2Theta:0.001; Minimum step size Omega:0.001<br>Sample stage=PW3071/xx Bracket<br>Diffractometer system=XPERT-PRO<br>Measurement program=Theta (10-90), Owner=User-1, Creation date=1/25/2018 8:59:22 AM<br>0.02 degpermin 46 min
Measurement Date / Time:	2/20/2020 12:32:58 PM
Operator:	State Univ of Malang
Raw Data Origin:	XRD measurement (*.XRDML)
Scan Axis:	Gonio
Start Position [°2Th.]:	10.0100
End Position [°2Th.]:	89.9900
Step Size [°2Th.]:	0.0200
Scan Step Time [s]:	0.7000
Scan Type:	Continuous
Offset [°2Th.]:	0.0000
Divergence Slit Type:	Fixed
Divergence Slit Size [°]:	0.9570
Specimen Length [mm]:	10.00
Receiving Slit Size [mm]:	0.1000
Measurement Temperature [°C]:	25.00
Anode Material:	Cu
K-Alpha1 [Å]:	1.54060
K-Alpha2 [Å]:	1.54443
K-Beta [Å]:	1.39225
K-A2 / K-A1 Ratio:	0.50000
Generator Settings:	35 mA, 40 kV
Diffractometer Type:	0000000011063758
Diffractometer Number:	0
Goniometer Radius [mm]:	240.00
Dist. Focus-Diverg. Slit [mm]:	91.00
Incident Beam Monochromator:	No
Spinning:	No

Graphics

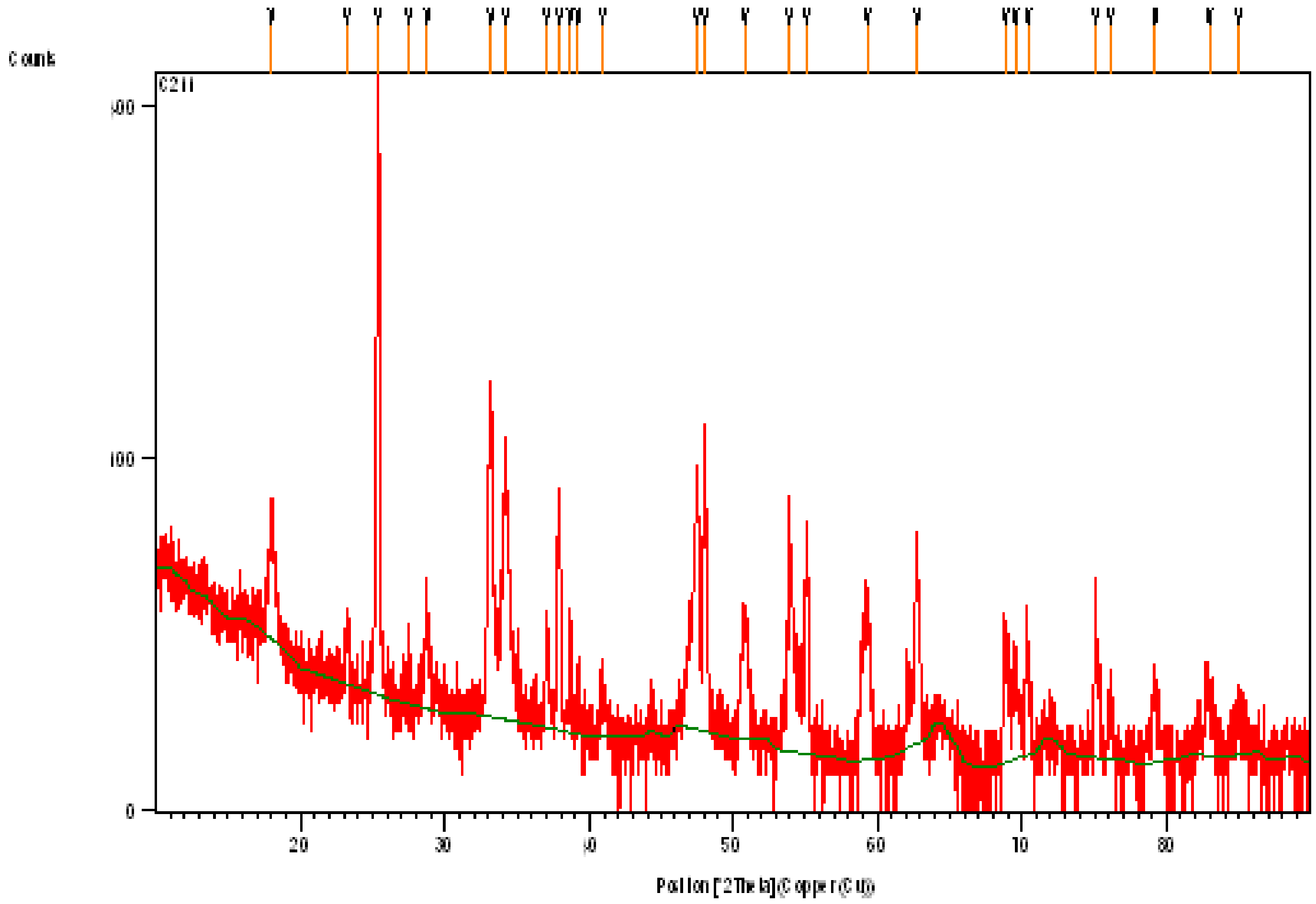

Peak List

Pos.[°2Th.]	Height[cts]	FWHM[°2Th.]	d-spacing[Å]	Rel.Int.[%]
18.0205	52.46	0.2755	4.92262	12.19
23.2288	11.86	0.4723	3.82934	2.76
25.3564	430.37	0.1378	3.51265	100.00
27.4611	13.02	0.2362	3.24801	3.03
28.7277	24.75	0.3149	3.10763	5.75
33.1593	126.79	0.2362	2.70174	29.46
34.1524	88.63	0.3149	2.62542	20.59
37.0163	24.51	0.1574	2.42861	5.69
37.8662	75.62	0.1378	2.37603	17.57
38.6335	21.36	0.1968	2.33059	4.96
39.1665	10.65	0.2362	2.30010	2.47
40.8899	7.46	0.4723	2.20704	1.73
47.5693	76.79	0.2362	1.91157	17.84
48.1004	107.82	0.2362	1.89170	25.05
50.8915	26.63	0.5510	1.79431	6.19
53.9459	70.41	0.1968	1.69971	16.36
55.1351	61.67	0.1968	1.66583	14.33
59.3225	30.61	0.3149	1.55785	7.11
62.7570	52.09	0.1181	1.48061	12.10
68.8743	16.99	0.3149	1.36328	3.95
69.5259	16.82	0.3149	1.35209	3.91
70.3905	17.64	0.3149	1.33759	4.10
75.0918	34.62	0.1181	1.26508	8.04
76.1397	6.64	0.4723	1.25026	1.54
79.2405	8.67	0.4723	1.20894	2.01
82.9070	6.90	0.6298	1.16454	1.60
84.9902	4.78	0.9600	1.14029	1.11

Document History

Insert Measurement:
- File name = "C211.xrdml"
- Modification time = "2/20/2020 2:40:38 PM"
- Modification editor = "State Univ of Malang"

Default properties:
- Measurement step axis = "None"
- Internal wavelengths used from anode material: Copper (Cu)
- Original K-Alpha1 wavelength = "1.54060"
- Used K-Alpha1 wavelength = "1.54060"
- Original K-Alpha2 wavelength = "1.54443"
- Used K-Alpha2 wavelength = "1.54443"
- Original K-Beta wavelength = "1.39225"
- Used K-Beta wavelength = "1.39225"
- Dist. focus to div. slit = "91.00000"
- Irradiated length = "10.00000"
- Spinner used = "No"
- Linear detector mode = "None"
- Length linear detector = "2"
- Step axis value = "0.00000"
- Offset = "0.00000"
- Sample length = "10.00000"
- Modification time = "2/20/2020 2:40:38 PM"
- Modification editor = "State Univ of Malang"

Search Peaks:
- Minimum significance = "2.00"
- Minimum tip width = "0.01"
- Maximum tip width = "1.00"
- Peak base width = "2.00"
- Method = "Top of smoothed peak"
- Modification time = "4/17/2017 8:55:59 AM"
- Modification editor = "State Univ of Malang"
