# Supplementary material for: Experimental data of CaTiO3 photocatalyst for degradation of organic pollutants (Brilliant green dye) – Green synthesis, characterization and kinetic study
Source: Data Brief. 2020 Jul 31;32:106099. doi: 10.1016/j.dib.2020.106099 (PMC7451799; doi:10.1016/j.dib.2020.106099)
Supplement: Supplementary file 1 [file mmc1.zip › All RAW Data for Data in Brief/XRD/XRD_CaTiO3 (1_3).pdf]

Anchor Scan Parameters

Dataset Name:	C212
File name:	E:\X'Pert Data\2020\Februari\20Jan 2020\C212\C212.xrdml
Sample Identification:	C212
Comment:	Theta (10-90)<br>Configuration=Stage Flat Samples, Owner=User-1, Creation date=9/15/2009 2:20:30 PM<br>Goniometer=PW3050/60 (Theta/Theta); Minimum step size 2Theta:0.001; Minimum step size Omega:0.001<br>Sample stage=PW3071/xx Bracket<br>Diffractometer system=XPERT-PRO<br>Measurement program=Theta (10-90), Owner=User-1, Creation date=1/25/2018 8:59:22 AM<br>0.02 degpermin 46 min
Measurement Date / Time:	2/20/2020 1:24:39 PM
Operator:	State Univ of Malang
Raw Data Origin:	XRD measurement (*.XRDML)
Scan Axis:	Gonio
Start Position [°2Th.]:	10.0100
End Position [°2Th.]:	89.9900
Step Size [°2Th.]:	0.0200
Scan Step Time [s]:	0.7000
Scan Type:	Continuous
Offset [°2Th.]:	0.0000
Divergence Slit Type:	Fixed
Divergence Slit Size [°]:	0.9570
Specimen Length [mm]:	10.00
Receiving Slit Size [mm]:	0.1000
Measurement Temperature [°C]:	25.00
Anode Material:	Cu
K-Alpha1 [Å]:	1.54060
K-Alpha2 [Å]:	1.54443
K-Beta [Å]:	1.39225
K-A2 / K-A1 Ratio:	0.50000
Generator Settings:	35 mA, 40 kV
Diffractometer Type:	0000000011063758
Diffractometer Number:	0
Goniometer Radius [mm]:	240.00
Dist. Focus-Diverg. Slit [mm]:	91.00
Incident Beam Monochromator:	No
Spinning:	No

Graphics

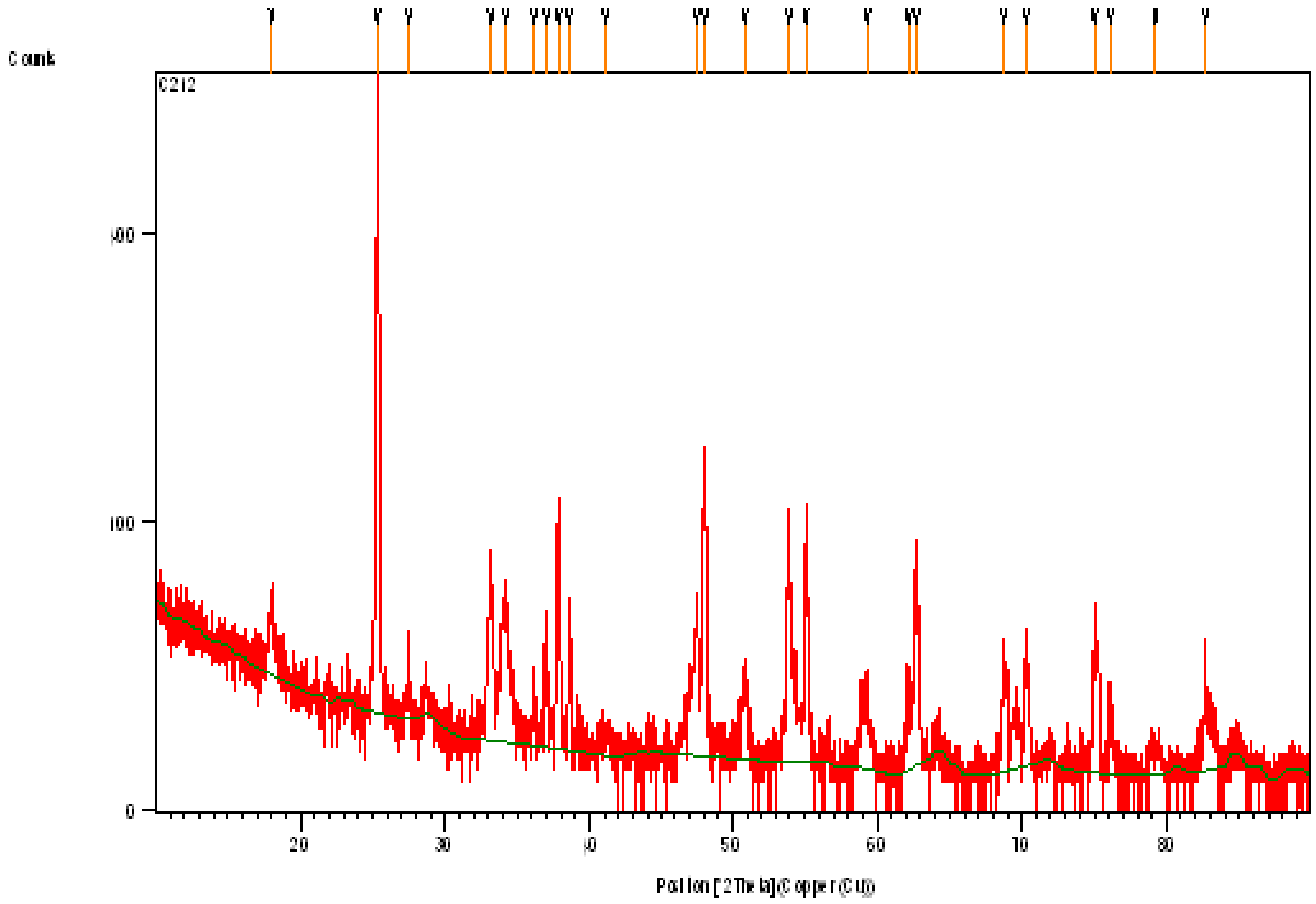

Peak List

Pos.[°2Th.]	Height[cts]	FWHM[°2Th.]	d-spacing[Å]	Rel.Int.[%]
17.9784	32.94	0.3149	4.93404	5.09
25.3334	647.01	0.1378	3.51577	100.00
27.4432	26.38	0.1574	3.25010	4.08
33.1247	76.21	0.1574	2.70449	11.78
34.1360	47.34	0.4330	2.62664	7.32
36.1262	13.10	0.2362	2.48638	2.02
36.9934	37.98	0.1968	2.43006	5.87
37.8224	112.45	0.1378	2.37868	17.38
38.5913	36.71	0.1968	2.33304	5.67
41.0367	2.75	0.9446	2.19949	0.42
47.5473	42.98	0.2755	1.91241	6.64
48.0665	145.21	0.1378	1.89295	22.44
50.8620	18.35	0.3149	1.79528	2.84
53.9034	104.32	0.1378	1.70095	16.12
55.0860	99.20	0.0984	1.66720	15.33
59.2976	18.76	0.2362	1.55845	2.90
62.1642	16.06	0.2362	1.49329	2.48
62.7071	70.96	0.1968	1.48167	10.97
68.7557	28.21	0.1574	1.36534	4.36
70.2953	30.91	0.1181	1.33916	4.78
75.0575	45.80	0.1181	1.26558	7.08
76.1019	9.94	0.2362	1.25079	1.54
79.2005	3.91	0.7872	1.20945	0.60
82.6835	28.06	0.1920	1.16615	4.34

Document History

Insert Measurement:
- File name = "C212.xrdml"
- Modification time = "2/20/2020 2:41:09 PM"
- Modification editor = "State Univ of Malang"

Default properties:
- Measurement step axis = "None"
- Internal wavelengths used from anode material: Copper (Cu)
- Original K-Alpha1 wavelength = "1.54060"
- Used K-Alpha1 wavelength = "1.54060"
- Original K-Alpha2 wavelength = "1.54443"
- Used K-Alpha2 wavelength = "1.54443"
- Original K-Beta wavelength = "1.39225"
- Used K-Beta wavelength = "1.39225"
- Dist. focus to div. slit = "91.00000"
- Irradiated length = "10.00000"
- Spinner used = "No"
- Linear detector mode = "None"
- Length linear detector = "2"
- Step axis value = "0.00000"
- Offset = "0.00000"
- Sample length = "10.00000"
- Modification time = "2/20/2020 2:41:09 PM"
- Modification editor = "State Univ of Malang"

Search Peaks:
- Minimum significance = "2.00"
- Minimum tip width = "0.01"
- Maximum tip width = "1.00"
- Peak base width = "2.00"
- Method = "Top of smoothed peak"
- Modification time = "4/17/2017 8:55:59 AM"
- Modification editor = "State Univ of Malang"
