# Supplementary material for: Experimental data of CaTiO3 photocatalyst for degradation of organic pollutants (Brilliant green dye) – Green synthesis, characterization and kinetic study
Source: Data Brief. 2020 Jul 31;32:106099. doi: 10.1016/j.dib.2020.106099 (PMC7451799; doi:10.1016/j.dib.2020.106099)
Supplement: Supplementary file 1 [file mmc1.zip › All RAW Data for Data in Brief/XRD/XRD_CaTiO3 (2_5).pdf]

Anchor Scan Parameters

Dataset Name:	C442
File name:	E:\X'Pert Data\2020\April\3 Apr 2020\C442\C442.xrdml
Sample Identification:	C442
Comment:	Theta (10-90)<br>Configuration=Stage Flat Samples, Owner=User-1, Creation date=9/15/2009 2:20:30 PM<br>Goniometer=PW3050/60 (Theta/Theta); Minimum step size 2Theta:0.001; Minimum step size Omega:0.001<br>Sample stage=PW3071/xx Bracket<br>Diffractometer system=XPERT-PRO<br>Measurement program=Theta (10-90), Owner=User-1, Creation date=1/25/2018 8:59:22 AM<br>0.02 degpermin 46 min
Measurement Date / Time:	4/3/2020 8:52:36 AM
Operator:	State Univ of Malang
Raw Data Origin:	XRD measurement (*.XRDML)
Scan Axis:	Gonio
Start Position [°2Th.]:	10.0100
End Position [°2Th.]:	89.9900
Step Size [°2Th.]:	0.0200
Scan Step Time [s]:	0.7000
Scan Type:	Continuous
Offset [°2Th.]:	0.0000
Divergence Slit Type:	Fixed
Divergence Slit Size [°]:	0.9570
Specimen Length [mm]:	10.00
Receiving Slit Size [mm]:	0.1000
Measurement Temperature [°C]:	25.00
Anode Material:	Cu
K-Alpha1 [Å]:	1.54060
K-Alpha2 [Å]:	1.54443
K-Beta [Å]:	1.39225
K-A2 / K-A1 Ratio:	0.50000
Generator Settings:	35 mA, 40 kV
Diffractometer Type:	0000000011063758
Diffractometer Number:	0
Goniometer Radius [mm]:	240.00
Dist. Focus-Diverg. Slit [mm]:	91.00
Incident Beam Monochromator:	No
Spinning:	No

Graphics

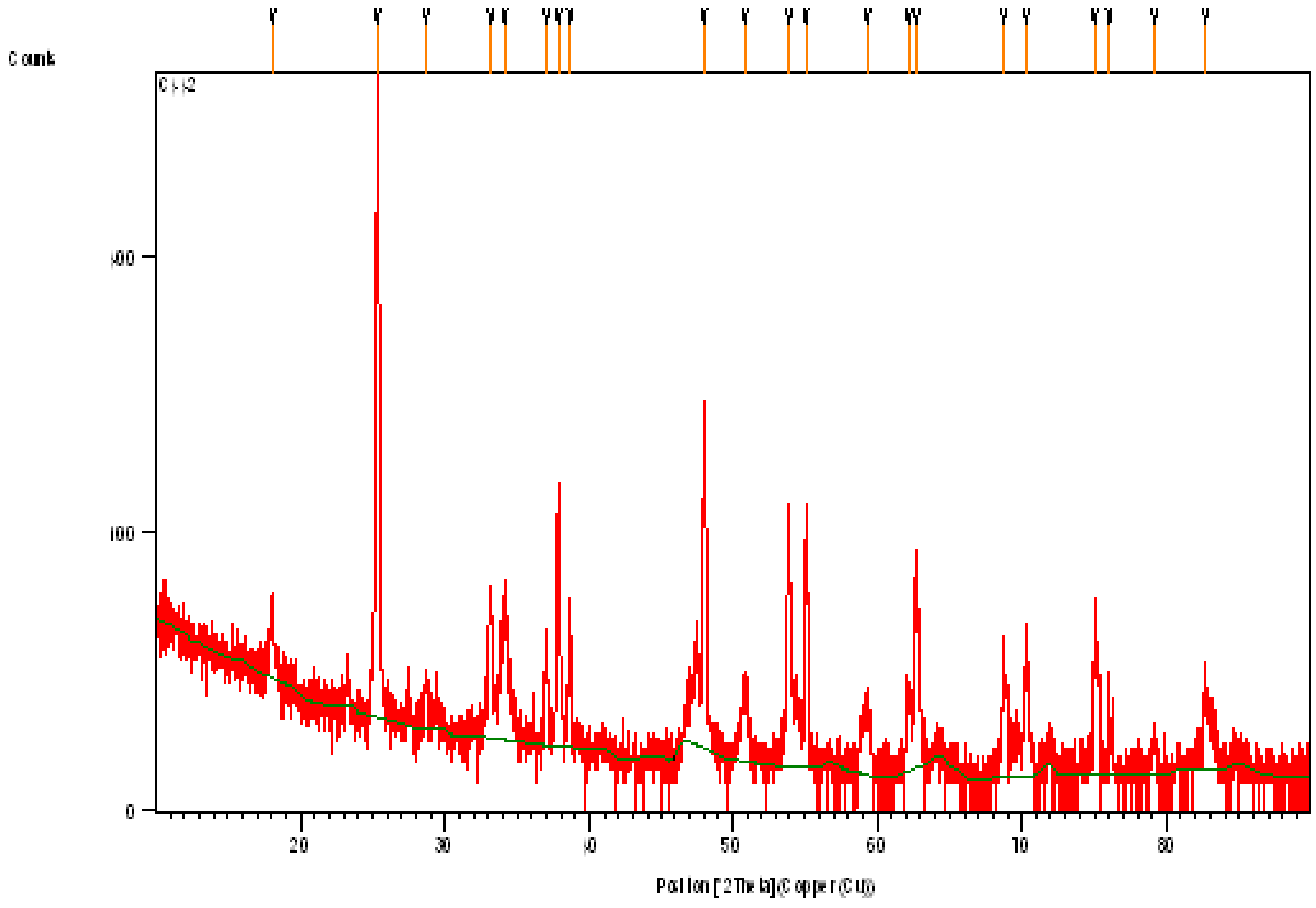

Peak List

Pos.[°2Th.]	Height[cts]	FWHM[°2Th.]	d-spacing[Å]	Rel.Int.[%]
18.0366	31.53	0.3149	4.91827	4.51
25.3200	699.32	0.1771	3.51761	100.00
28.6713	11.38	0.4723	3.11361	1.63
33.1325	55.41	0.2755	2.70387	7.92
34.0954	55.76	0.2362	2.62968	7.97
36.9818	35.06	0.1574	2.43080	5.01
37.8128	136.53	0.1378	2.37927	19.52
38.5978	41.68	0.1968	2.33267	5.96
48.0603	207.81	0.0984	1.89318	29.72
50.8590	17.43	0.3936	1.79538	2.49
53.8872	121.70	0.0720	1.70001	17.40
55.0776	119.99	0.0787	1.66743	17.16
59.2970	13.44	0.3149	1.55846	1.92
62.1840	15.85	0.2362	1.49287	2.27
62.6983	74.53	0.1181	1.48185	10.66
68.7623	34.05	0.1181	1.36523	4.87
70.3079	35.05	0.1574	1.33895	5.01
75.0555	51.78	0.1181	1.26560	7.40
76.0299	16.13	0.1181	1.25179	2.31
79.1440	4.19	0.4723	1.21017	0.60
82.6718	20.70	0.1920	1.16628	2.96

Document History

Insert Measurement:
- File name = "C442.xrdml"
- Modification time = "4/3/2020 9:40:13 AM"
- Modification editor = "State Univ of Malang"

Default properties:
- Measurement step axis = "None"
- Internal wavelengths used from anode material: Copper (Cu)
- Original K-Alpha1 wavelength = "1.54060"
- Used K-Alpha1 wavelength = "1.54060"
- Original K-Alpha2 wavelength = "1.54443"
- Used K-Alpha2 wavelength = "1.54443"
- Original K-Beta wavelength = "1.39225"
- Used K-Beta wavelength = "1.39225"
- Dist. focus to div. slit = "91.00000"
- Irradiated length = "10.00000"
- Spinner used = "No"
- Linear detector mode = "None"
- Length linear detector = "2"
- Step axis value = "0.00000"
- Offset = "0.00000"
- Sample length = "10.00000"
- Modification time = "4/3/2020 9:40:13 AM"
- Modification editor = "State Univ of Malang"

Search Peaks:
- Minimum significance = "2.00"
- Minimum tip width = "0.01"
- Maximum tip width = "1.00"
- Peak base width = "2.00"
- Method = "Top of smoothed peak"
- Modification time = "4/17/2017 8:55:59 AM"
- Modification editor = "State Univ of Malang"
