# Supplementary material for: Experimental data of CaTiO3 photocatalyst for degradation of organic pollutants (Brilliant green dye) – Green synthesis, characterization and kinetic study
Source: Data Brief. 2020 Jul 31;32:106099. doi: 10.1016/j.dib.2020.106099 (PMC7451799; doi:10.1016/j.dib.2020.106099)
Supplement: Supplementary file 1 [file mmc1.zip › All RAW Data for Data in Brief/XRD/XRD_CaTiO3 (2_7).pdf]

Anchor Scan Parameters

Dataset Name:	C443
File name:	E:\X'Pert Data\2020\April\3 Apr 2020\C443\C443.xrdml
Sample Identification:	C443
Comment:	Theta (10-90)<br>Configuration=Stage Flat Samples, Owner=User-1, Creation date=9/15/2009 2:20:30 PM<br>Goniometer=PW3050/60 (Theta/Theta); Minimum step size 2Theta:0.001; Minimum step size Omega:0.001<br>Sample stage=PW3071/xx Bracket<br>Diffractometer system=XPERT-PRO<br>Measurement program=Theta (10-90), Owner=User-1, Creation date=1/25/2018 8:59:22 AM<br>0.02 degpermin 46 min
Measurement Date / Time:	4/3/2020 8:01:41 AM
Operator:	State Univ of Malang
Raw Data Origin:	XRD measurement (*.XRDML)
Scan Axis:	Gonio
Start Position [°2Th.]:	10.0100
End Position [°2Th.]:	89.9900
Step Size [°2Th.]:	0.0200
Scan Step Time [s]:	0.7000
Scan Type:	Continuous
Offset [°2Th.]:	0.0000
Divergence Slit Type:	Fixed
Divergence Slit Size [°]:	0.9570
Specimen Length [mm]:	10.00
Receiving Slit Size [mm]:	0.1000
Measurement Temperature [°C]:	25.00
Anode Material:	Cu
K-Alpha1 [Å]:	1.54060
K-Alpha2 [Å]:	1.54443
K-Beta [Å]:	1.39225
K-A2 / K-A1 Ratio:	0.50000
Generator Settings:	35 mA, 40 kV
Diffractometer Type:	0000000011063758
Diffractometer Number:	0
Goniometer Radius [mm]:	240.00
Dist. Focus-Diverg. Slit [mm]:	91.00
Incident Beam Monochromator:	No
Spinning:	No

Graphics

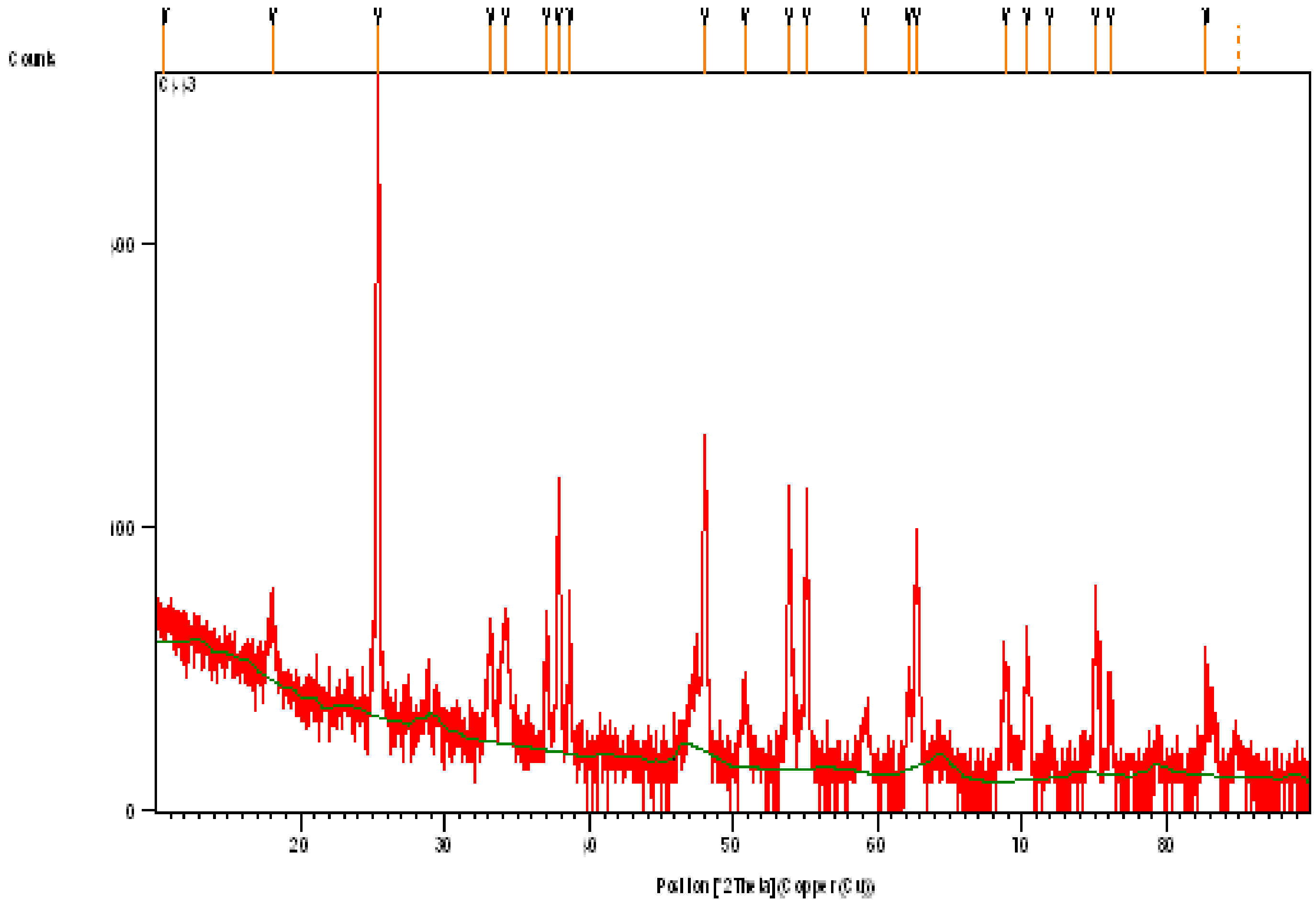

Peak List

Pos.[°2Th.]	Height[cts]	FWHM[°2Th.]	d-spacing[Å]	Rel.Int.[%]
10.5897	9.68	0.9446	8.35425	1.51
18.0621	31.01	0.3149	4.91137	4.85
25.3490	639.83	0.1574	3.51365	100.00
33.1390	33.68	0.2755	2.70336	5.26
34.1745	39.65	0.3149	2.62377	6.20
37.0032	34.49	0.1968	2.42944	5.39
37.8311	133.72	0.0787	2.37816	20.90
38.5958	47.95	0.1181	2.33278	7.49
48.0992	172.17	0.1181	1.89174	26.91
50.8760	17.19	0.2755	1.79482	2.69
53.9360	108.54	0.2362	1.70000	16.96
55.1172	115.38	0.0984	1.66633	18.03
59.2194	8.06	0.6298	1.56032	1.26
62.1973	15.85	0.2362	1.49258	2.48
62.7311	86.97	0.1181	1.48116	13.59
68.8382	27.37	0.3149	1.36391	4.28
70.3300	40.89	0.1378	1.33859	6.39
71.8881	3.15	0.4723	1.31336	0.49
75.0837	59.65	0.1181	1.26520	9.32
76.0742	17.20	0.1181	1.25117	2.69
82.7050	25.18	0.1440	1.16590	3.94
84.9626	2.63	1.1520	1.14343	0.41

Document History

Insert Measurement:
- File name = "C443.xrdml"
- Modification time = "4/3/2020 9:35:17 AM"
- Modification editor = "State Univ of Malang"

Default properties:
- Measurement step axis = "None"
- Internal wavelengths used from anode material: Copper (Cu)
- Original K-Alpha1 wavelength = "1.54060"
- Used K-Alpha1 wavelength = "1.54060"
- Original K-Alpha2 wavelength = "1.54443"
- Used K-Alpha2 wavelength = "1.54443"
- Original K-Beta wavelength = "1.39225"
- Used K-Beta wavelength = "1.39225"
- Dist. focus to div. slit = "91.00000"
- Irradiated length = "10.00000"
- Spinner used = "No"
- Linear detector mode = "None"
- Length linear detector = "2"
- Step axis value = "0.00000"
- Offset = "0.00000"
- Sample length = "10.00000"
- Modification time = "4/3/2020 9:35:17 AM"
- Modification editor = "State Univ of Malang"

Search Peaks:
- Minimum significance = "2.00"
- Minimum tip width = "0.01"
- Maximum tip width = "1.00"
- Peak base width = "2.00"
- Method = "Top of smoothed peak"
- Modification time = "4/17/2017 8:55:59 AM"
- Modification editor = "State Univ of Malang"
