# Supplementary figures and images for: Experimental data of CaTiO3 photocatalyst for degradation of organic pollutants (Brilliant green dye) – Green synthesis, characterization and kinetic study
Source: Data Brief. 2020 Jul 31;32:106099. doi: 10.1016/j.dib.2020.106099 (PMC7451799; doi:10.1016/j.dib.2020.106099)

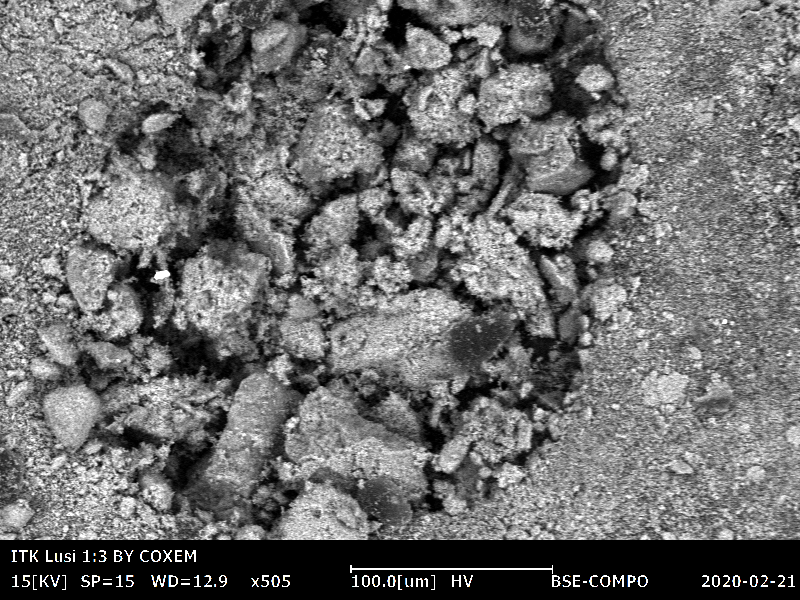

Supplement: Supplementary file 1 [file mmc1.zip › All RAW Data for Data in Brief/SEM/SEM CaTiO3 (1_1) _ 1st.bmp]

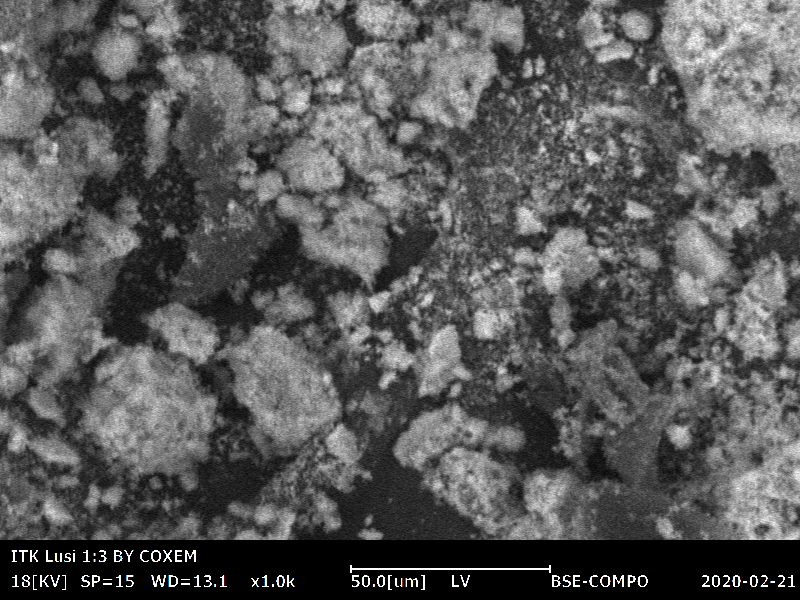

Supplement: Supplementary file 1 [file mmc1.zip › All RAW Data for Data in Brief/SEM/SEM CaTiO3 (1_1) _ 2nd.bmp]

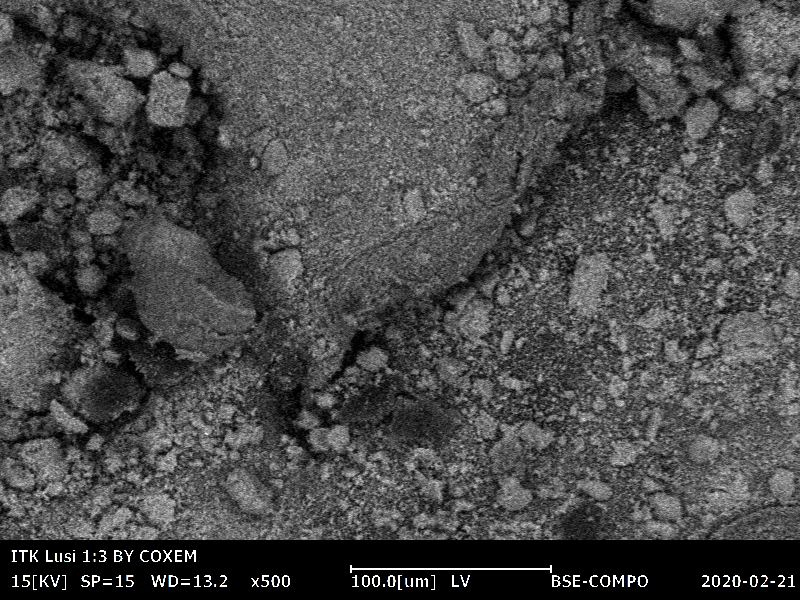

Supplement: Supplementary file 1 [file mmc1.zip › All RAW Data for Data in Brief/SEM/SEM CaTiO3 (1_1) _ 3rd.bmp]

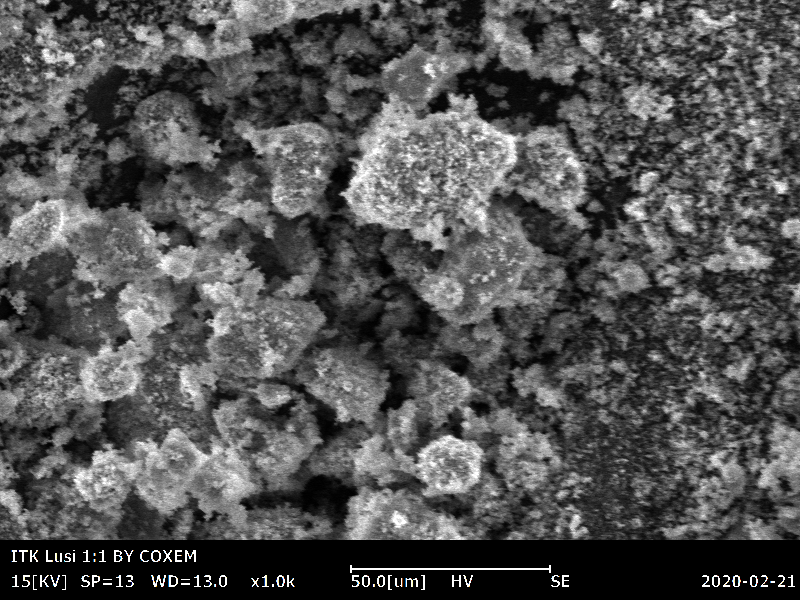

Supplement: Supplementary file 1 [file mmc1.zip › All RAW Data for Data in Brief/SEM/SEM CaTiO3 (1_3) _ 1st.bmp]

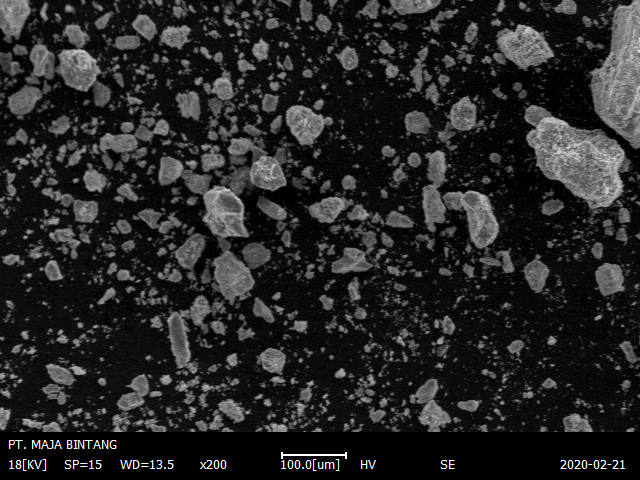

Supplement: Supplementary file 1 [file mmc1.zip › All RAW Data for Data in Brief/SEM/SEM CaTiO3 (1_3) _ 2nd.bmp]

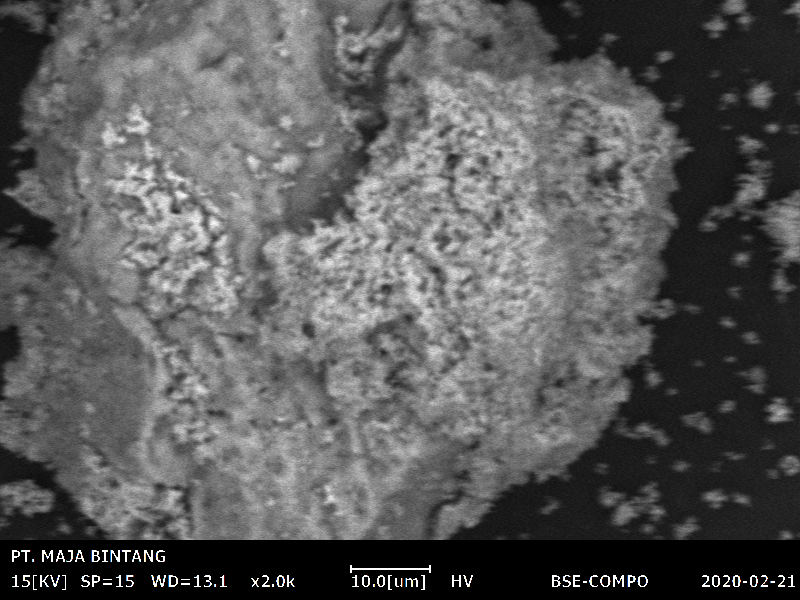

Supplement: Supplementary file 1 [file mmc1.zip › All RAW Data for Data in Brief/SEM/SEM CaTiO3 (1_3) _ 3rd.bmp]

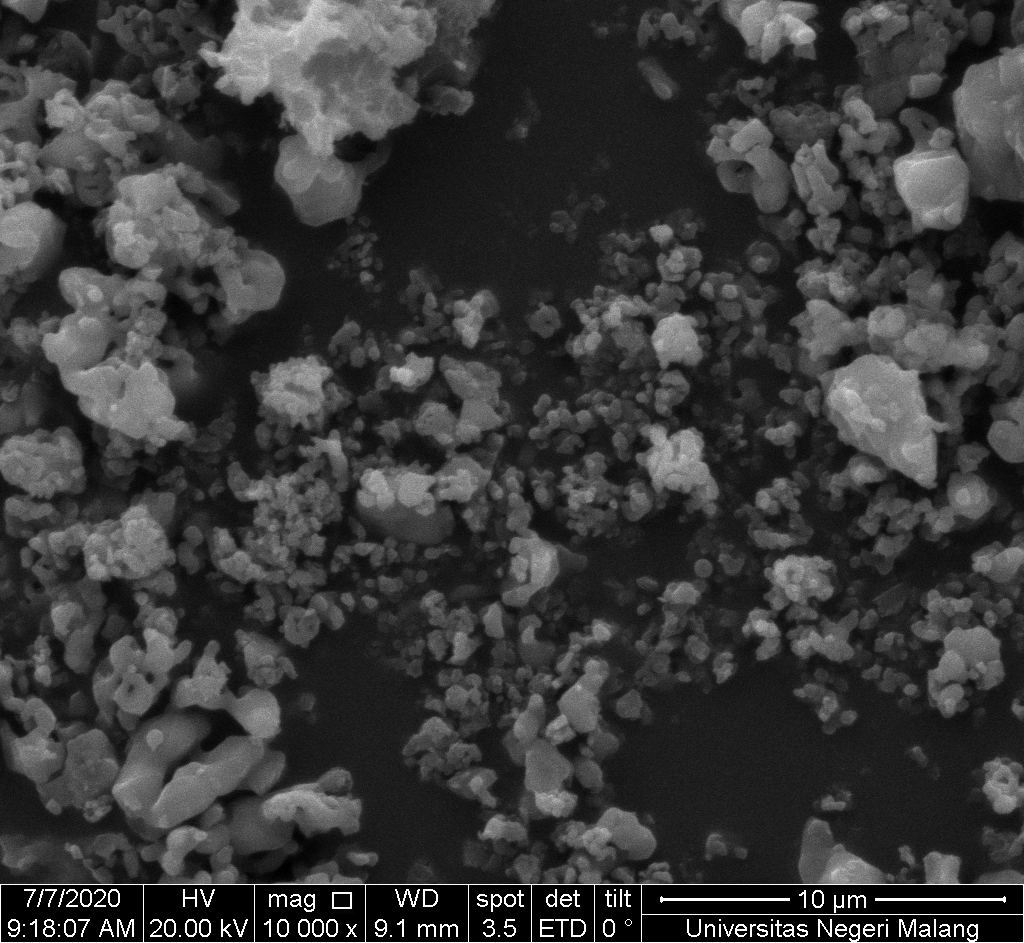

Supplement: Supplementary file 1 [file mmc1.zip › All RAW Data for Data in Brief/SEM/SEM CaTiO3 (2_5) _ 10kx.tif]

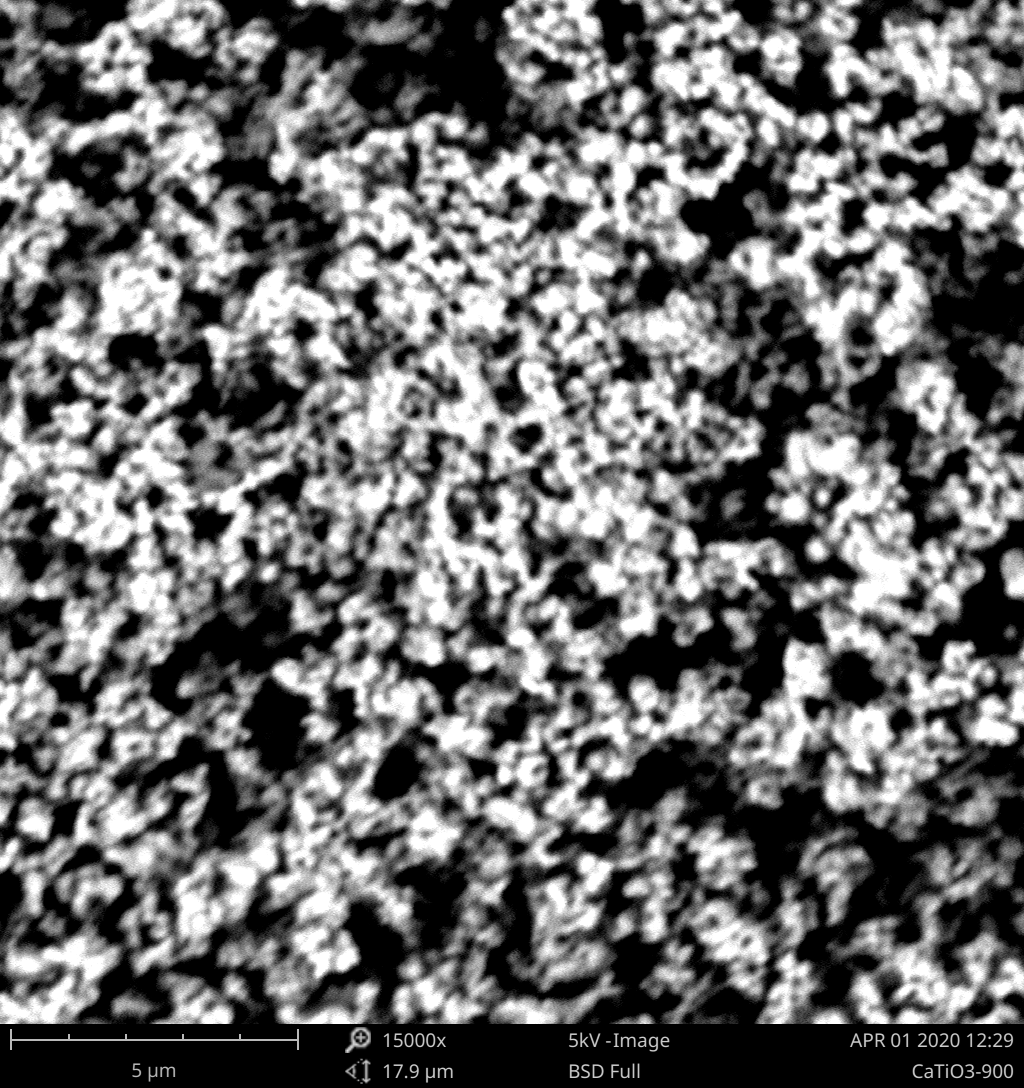

Supplement: Supplementary file 1 [file mmc1.zip › All RAW Data for Data in Brief/SEM/SEM CaTiO3 (2_7) _ 20kx.tiff]

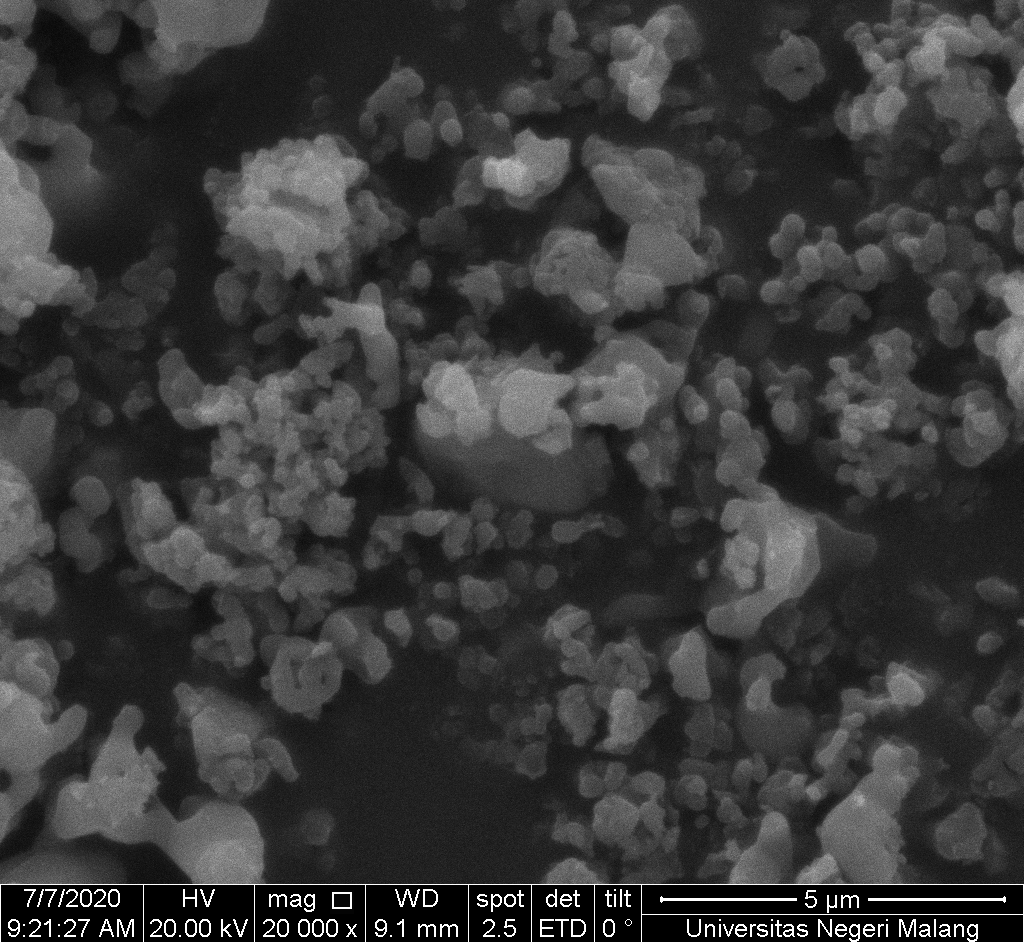

Supplement: Supplementary file 1 [file mmc1.zip › All RAW Data for Data in Brief/SEM/SEM_CaTiO3 (2_5) _ 20kx.tif]

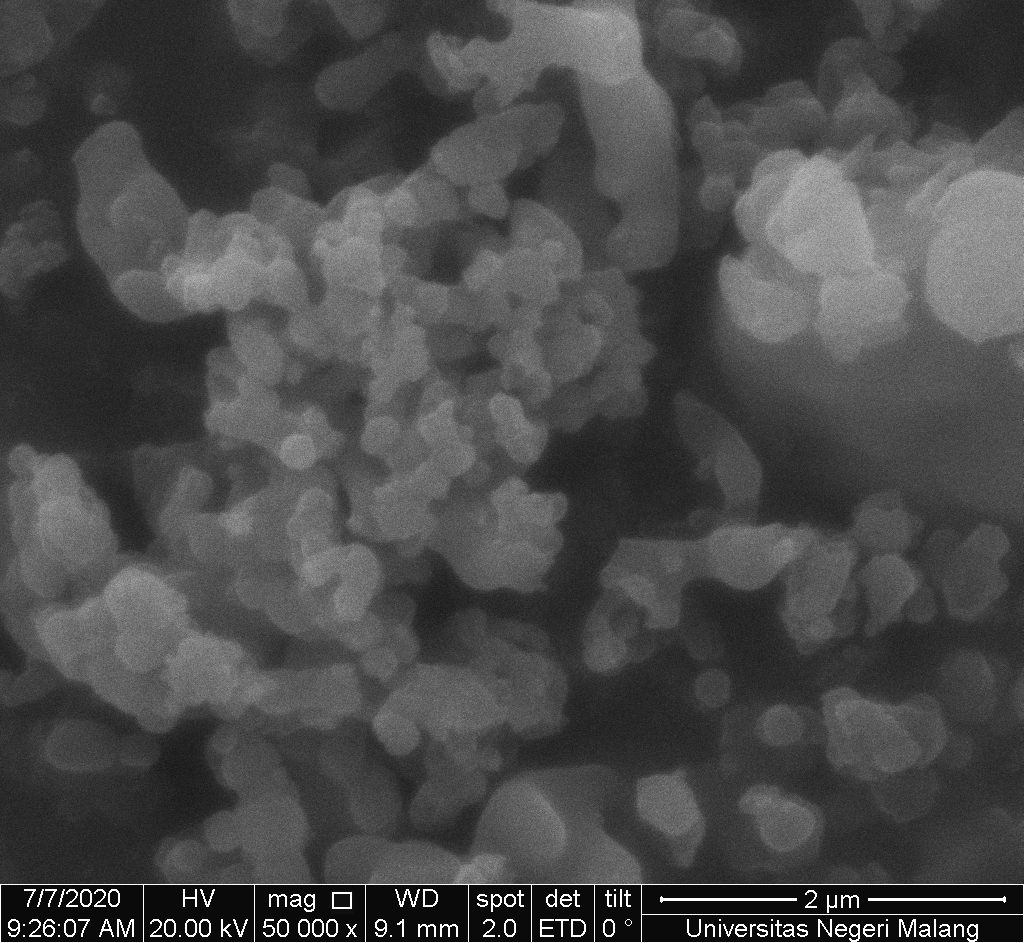

Supplement: Supplementary file 1 [file mmc1.zip › All RAW Data for Data in Brief/SEM/SEM_CaTiO3 (2_5) _ 50kx.tif]

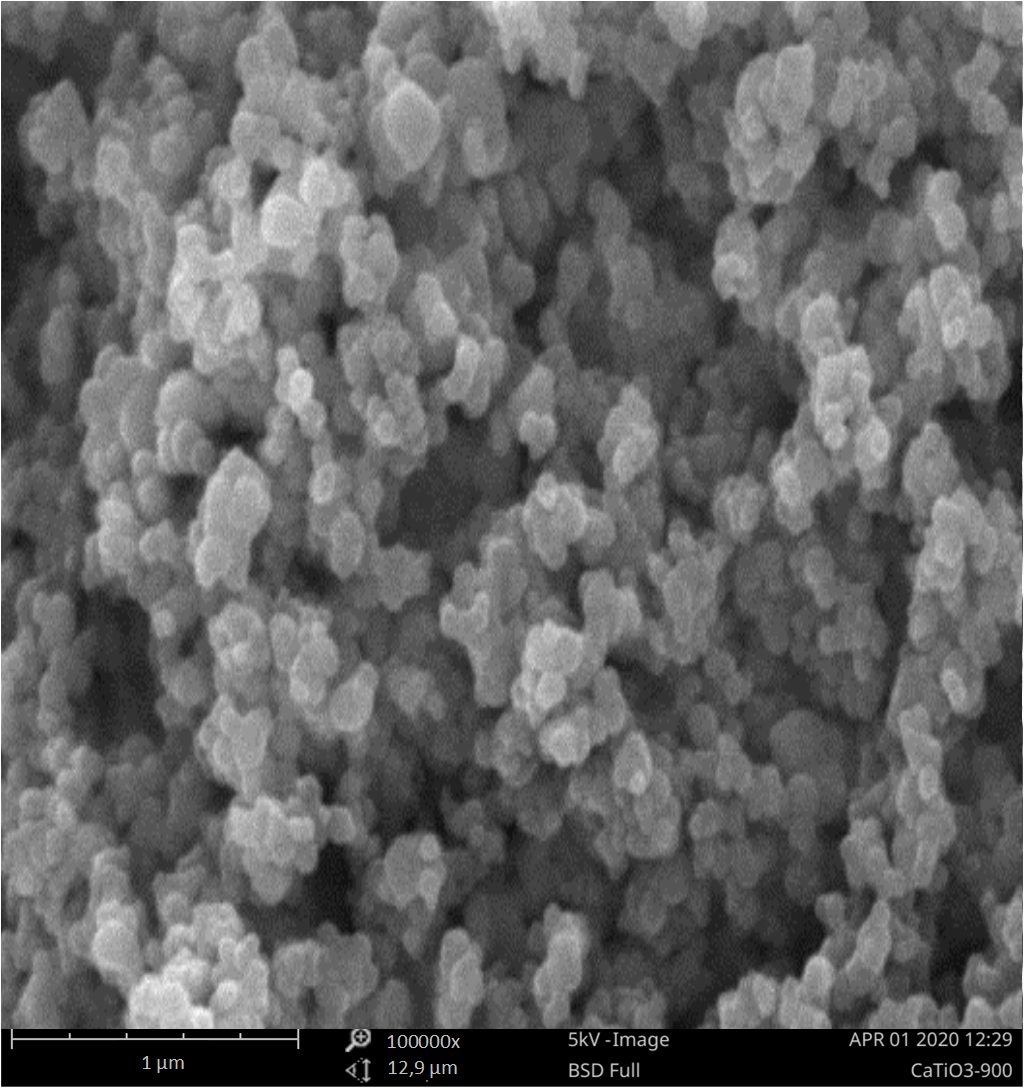

Supplement: Supplementary file 1 [file mmc1.zip › All RAW Data for Data in Brief/SEM/SEM_CaTiO3 (2_7) _ 100kx.tif]

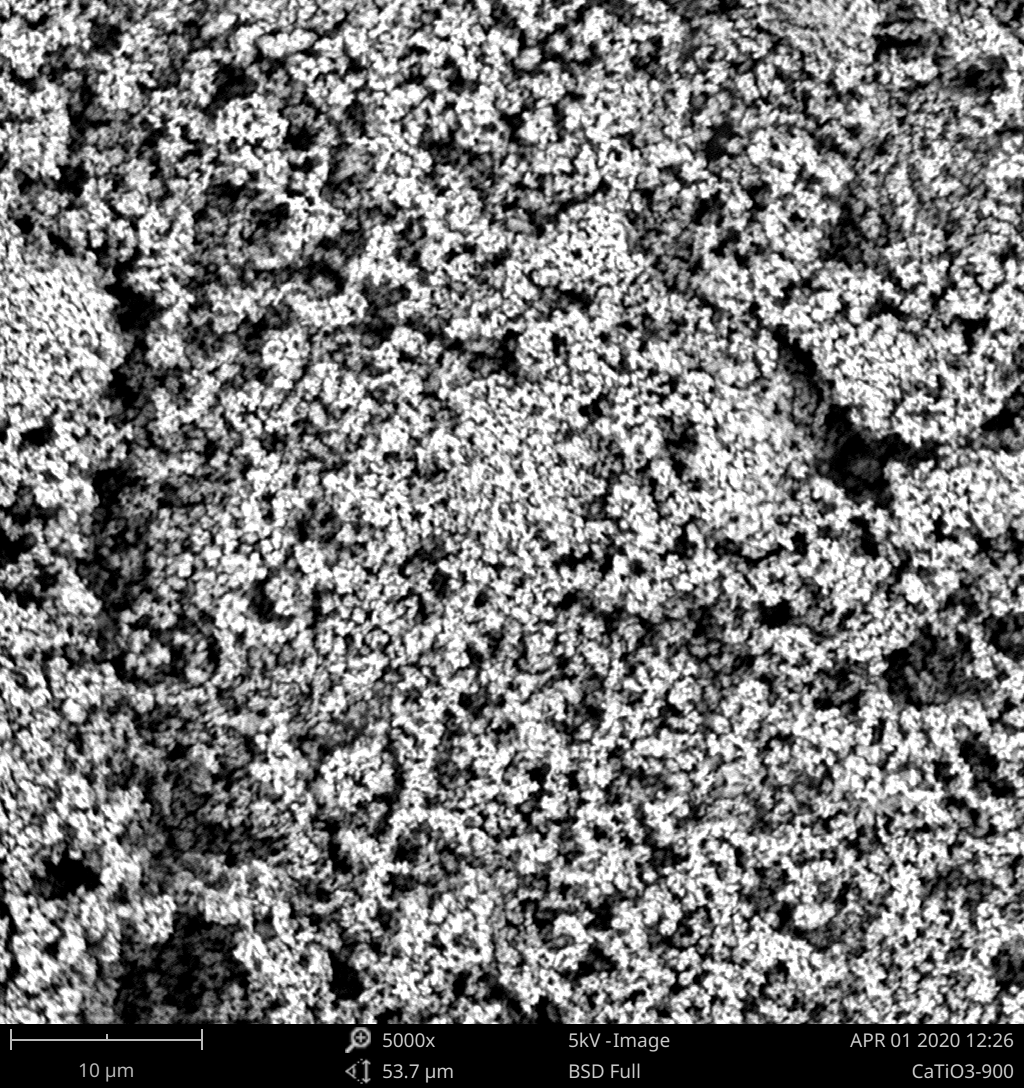

Supplement: Supplementary file 1 [file mmc1.zip › All RAW Data for Data in Brief/SEM/SEM_CaTiO3 (2_7) _ 10kx.tiff]
